# Epoxidized Soybean Oleic Acid/Oligomeric Poly(lactic acid)-Grafted Nano-Hydroxyapatite and Its Role as a Filler in Poly(L-lactide) for Potential Bone Fixation Application

**DOI:** 10.3390/ma17112620

**Published:** 2024-05-29

**Authors:** Chen Huang, Xin-Yu Luo, Zi-Sheng Chao, Yue-Fei Zhang, Kun Liu, Wen-Jun Yi, Li-Jun Li, Zeyan Zhou

**Affiliations:** 1 College of Materials Science and Engineering, Changsha University of Science & Technology, Changsha 410082, China; 19375178158@163.com (C.H.); 19308467138@163.com (X.-Y.L.); chao_zs@aliyun.com (Z.-S.C.); zhangyuefei@csust.edu.cn (Y.-F.Z.); 2 College of Chemistry and Chemical Engineering, Hunan Institute of Science and Technology, Yueyang 414006, China; liukun328@126.com; 3 College of Materials Science and Engineering, Hunan University, Changsha 410012, China

**Keywords:** epoxidized soybean oleic acid, hydroxyapatite, surface modification, oligomeric poly(L-lactic acid), biocompatibility, mechanical properties

## Abstract

One of the most effective strategies for modifying the surface properties of nano-fillers and enhancing their composite characteristics is through polymer grafting. In this study, a coprecipitation method was employed to modify hydroxyapatite (HAP) with epoxidized soybean oleic acid (ESOA), resulting in ESOA-HAP. Subsequently, oligomeric poly(lactic acid) (OPLA) was grafted onto the surface of ESOA-HAP, yielding OPLA-ESOA-HAP. HAP, ESOA-HAP, and OPLA-ESOA-HAP were comprehensively characterized. The results demonstrate the progressive grafting of ESOA and OPLA onto the surface of HAP, resulting in enhanced hydrophobicity and improved dispersity in organic solvent for OPLA-ESOA-HAP compared to HAP. The vitality and adhesion of Wistar rat mesenchymal stem cells (MSCs) were assessed using HAP and modified HAP materials. Following culture with MSCs for 72 h, the OPLA-ESOA-HAP showed an inhibition rate lower than 23.0% at a relatively high concentration (1.0 mg/mL), which is three times lower compared to HAP under similar condition. The cell number for OPLA-ESOA-HAP was 4.5 times higher compared to HAP, indicating its superior biocompatibility. Furthermore, the mechanical properties of the OPLA-ESOA-HAP/PLLA composite almost remained unaltered ever after undergoing two stages of thermal processing involving melt extrusion and inject molding. The increase in the biocompatibility and relatively high mechanical properties render OPLA-ESOA-HAP/PLLA a potential material for the biodegradable fixation system.

## 1. Introduction

In recent years, there has been a surge in the number of fractures, bone defects, and other related cases worldwide, leading to a rapid increase in patients with bone disease. Consequently, global resources for bone transplantation are becoming increasingly scarce while the incidence rate of bone transplant sites continues to rise [1]. The process of bone regeneration following transplantation is a complex, dynamic, and continuous one that commences with the migration of stem cells and subsequently involves proliferation, differentiates, and matrix deposition promotion, ultimately culminating in the self-repair of the bone tissue [2]. However, this regenerative capacity is inherently limited and necessitates exogenous assistance for the repair of large-sized defects. In the early stages of bone repair technology, autologous or homologous bone transplantation emerged as the predominant method due to its ability to minimize immune system rejection. However, there has been a subsequent significant increase in the incidence rate of autologous bone transplantation, resulting in a double burden for patients who have already suffered from bone disease. Bioabsorbable materials are widely regarded as an optimal choice for bone fixation and repair applications due to the metabolic degradation that occurs within the human body [3]. This characteristic helps obviate the necessity of additional surgery for metal implant removal. Poly(L-lactic acid) (PLLA) is a crucial material in bone regeneration applications for contemporary biomedical implants [4], owing to its exceptional mechanical stability, processability, cellular compatibility, and degradation properties that align with new bone formation. Furthermore, it degrades into non-toxic and metabolizable lactic acid [5].

The potential for PLLA application has been demonstrated in the fields of tissue engineering, suture, implantation, and drug delivery [6,7]. Despite extensive research on PLLA for its exceptional performance, its biological inertness restricts its application in bone repair engineering due to inadequate bone conductivity and bone induction. However, bioceramics such as hydroxyapatite (HAP) or β-tricalcium phosphate (β-TCP) are more commonly chosen as bone repair materials due to their ability to enhance bone osteoconductive and mitigate aseptic inflammation caused by the degradation product (L-lactic acid) [8,9]. The formation of HAP/PLLA composites is confronted with two significant challenges, namely the limited bearing capacity and the relatively low strength of the composite. These issues arise from two factors: the accumulation of HAP in the PLLA matrix and the weak interaction force between HAP and PLLA polymers. Those two factors affect the mechanical strength of composites prepared by electrostatic spinning [10,11], 3D printing, injection molding, and hot-press molding. The surface modification of HAP was conducted to enhance the interfacial adhesion between HAP and PLLA polymers, as well as to optimize the dispersion of HAP within the PLLA matrix. Coupling agents such as silane [12], lactic acid, and polymers are applied to tailor its surface properties; Additionally, research has demonstrated that graphene oxide grafted onto HAP can enhance its mechanical properties while preserving its biological attributes. However, these approaches may exhibit certain limitations, including biotoxicity or the requirement for high-temperature calcination, which could potentially impact the composite properties [13]. Among all these modification methods, polymer grafting is thought to be the most effective approach for enhancing the interaction between HAP and the PLLA matrix due to the ability of polymers to intricately intertwine with PLLA matrix.

In our previous work, the polymer-modified HAP exhibited superior performance compared to the unmodified HAP-filled PLLA composite prepared via hot-press molding. While the utilization of twin-screw extrusion and injection molding for bone repair material preparation is prevalent in the industry, the fragility of samples tend to increase after undergoing two stages of heat treatment. Therefore, our objective is to identify a coupling agent that can enhance both the distribution of HAP within the PLLA matrix and its interaction with PLLA. Epoxized soybean oil (ESBO) is derived from soybean oil and is known for its non-toxicity, metabolization, accessibility, and cost-effectiveness [14]. This oil can be applied to biological materials [15] and can be used as an effective plasticizer [16] for PLLA which may alter its processability. Research findings also indicate that the incorporation of ESBO into the PLA matrix leads to enhanced flexibility in plasticized PLA sheets, and as the ESBO content increases, there is a significant improvement in the mechanical properties of the plasticized PLA film. In this work, epoxized soybean oil acid (ESOA), which is derived from ESBO, is selected as a coupling agent for further surface modification of HAP. It is intended to act as a bridge between HAP and PLA matrices, enhancing their interface compatibility by utilizing the -COOH chelate with HAP and the oleophilic part of the ESOA that can interact with PLLA [17,18]. Consequently, the modification of HAP with ESOA provide additional active sites for subsequent OPLA grafting, which could further improve the hydrophobicity and biocampatibility of HAP. This synergistic approach enhances the mechanical properties of the HAP/PLLA composite, facilitating the development of superior bone repair materials that can be efficiently produced through injection molding.

## 2. Materials and Methods

### 2.1. Materials

Reactants: CaCl_2_, Na_3_PO_4_·12H_2_O, NaOH, and epoxidized soybean oil (ESBO) were purchased from Aladdin Chemical Reagent Company (Shanghai, China). Oligomeric poly(lactic acid) (OPLA) was synthesized following our previous work [19], with a viscosity average molecular weight of the OPLA being about 6000. Commercial Poly(L-lactide) (PLLA) was purchased, and the brand name was Natural Work 4032D.

Solvents: Xylene and CH_3_Cl were obtained from Sinopharm Chemical Reagent Company (Shanghai, China), dried using calcium dihydride, and then distilled under a reduced pressure.

### 2.2. Synthesis of the Materials

#### 2.2.1. Synthesis of Nano-Hydroxyapatite (HAP) and Epoxidized Soybean Oil-Grafted Hydroxyapatite (ESOA-HAP)

The aqueous CaCl_2_ solution (500 mL, 0.4 M) was adjusted to a pH range of 9.0–10.0 using the 0.1 M NaOH solution and subsequently heated at 60 °C. Then, 300 mL of a 0.3 M Na_3_PO_4_ solution was added dropwise to the aforementioned CaCl_2_ solution and stirred for 30 min. By adding an aqueous solution of 0.1 M NaOH, the pH of the reaction mixture was maintained within the range of 9.0 and 10.0. Subsequently, the solution was stirred at a temperature of 60 °C for a duration of 30 min, followed by an aging period of 24 h. The mixture undergoes filtration, followed by the repeated rinsing of the filtrate with deionized water five times to eliminate any non-grafted ESOA. Subsequently, the mixture was freeze-dried for a duration of 24 h. The resulting filtrate was designated as HAP.

The hydrolysis of 0.6 g epoxidized soybean oil was conducted in a NaOH alcohol-water solution to obtained neutralized epoxidized soybean oleic acid (Na-ESOA). Subsequently, it was mixed with a Na_3_PO_4_ (300 mL, 0.3 M) aqueous solution and co-precipitated with CaCl_2_ (500 mL 0.4 M) following the aforementioned procedure. The resulting filtrate was referred to as ESOA-HAP.

#### 2.2.2. Synthesis of OPLA-Grafted HAP (OPLA-HAP) and OPLA-Grafted ESOA-HAP (OPLA-ESOA-HAP)

Then, 1.0 g of HAP, 2.0 g of OPLA (M_η_ = ca. 6, 000), 0.02 g of triethylamine, and 20 mL of dried xylene were added into a flask. The resulting mixture was stirred and refluxed at a temperature of 130 °C for a duration of 24 h. The free OPLA was completely removed from the mixture through centrifugation and dichloromethane washing after it cooled to room temperature. Subsequently, the obtained solid was vacuum dried at 40 °C for 10 h. This resulting powder was designated as OPLA-HAP.

The ESOA-HAP was modified following the same procedures employed for the preparation of OPLA-HAP, thereby yielding a powder denoted as OPLA-ESOA-HAP.

#### 2.2.3. Preparation of the Blends Comprised of the HAP or Modified HAP with PLLA

The HAPs (HAP, ESOA-HAP, and OPLA-ESOA-HAP) were incorporated into PLLA to synthesize HAPs/PLLA blends through melt mixing and inject molding techniques. The composites containing 2.5%, 5.0%, and 7.5% of HAP, ESOA-HAP, and OPLA-ESOA-HAP were prepared, respectively. These composites were referred to as HAP/PLLA, ESOA-HAP/PLLA, and OPLA-ESOA-HAP/PLLA. The PLLA pellets and HAPs were subjected to vacuum drying at 80 °C for 3 h before compounding. A co-rotating twin-screw extruder (Giant SHJ-20) equipped with volumetric feeder was employed for the compounding of HAP/PLLA composites. The extruder was equipped with six controlled temperature sections, set as 150 °C, 160 °C, 180 °C, 190 °C, 190 °C, and 190 °C (die adaptor). Following air cooling, the extruded substance was granulated using a strand pelletizer. Composites molding was carried out using an injecting molding machine (Jinying GEK-80, Zhoushan, China). The obtained composites pellets undergo vacuum drying at 90 °C for a duration of 5 h before inject molding. The barrel temperature was set at 195 °C (feeding section), 190 °C, 185 °C, and 180 °C (nozzle). The mold temperature was 30 °C, mold time was 15 s, and mold pressure was 60 MPa. The molded sample was rapidly cooled to room temperature within a span of 30 s. PLLA strand pelletizer was prepared directly via injecting molding without the melt mixing process.

### 2.3. Characterizations

X-ray diffraction (XRD) experiments were conducted using a Bruker D8 Advance X-ray diffractometer under the following situations: Cu target Kα radiation (λ = 1.54187 Å); scanning voltage of 40 kV; scanning current of 40 mA; scanning speed of 60 min^−1^; scanning step of 0.02°.

Fourier-transform infrared spectroscopy (FT-IR) analysis was performed using a Varian 3100 instrument, encompassing a total of 32 scans, ranging from 400 cm^−1^ to 4000 cm^−1^, with a resolution of 2 cm^−1^, thereby generating the composite spectrum.

Thermal gravimetric analysis (TGA) was conducted using a TA SDT Q500 device, with air employed as the carrier gas. The sample underwent heating from ambient temperature to 800 °C at a rate of 10 °C·min^−1^.

Scanning electron microscopy (SEM) was performed on a JEOL JSM-6700F instrument operated at an accelerating voltage of 20 kV. Prior to characterization, the particle samples were dispersed in dichloromethane and deposited onto a silicon chip. To create a randomly brittle cracked surface, the composite samples were immersed in illiquid nitrogen for five minutes. Subsequently, all samples underwent uniform gold coating.

Differential scanning calorimeter (DSC) measurements were carried out using a Perkin Elmer DSC-7, with the temperature increased at a rate of 10 °C·min^−1^ from 20 °C to 180 °C, followed by a subsequent decrease at the same rate from 180 °C to 0 °C. Then, the temperature increased from 0 °C to 180 °C at a rate of 10 °C·min^−1^. The crystallinity of PLLA in the composites was determined using Equation (1).

X (%) = (ΔH_m_/93.7) × 100% (1)

X: crystallinity; ΔH_m_: the melting enthalpy (J·g^−1^). The theoretical enthalpy of completely crystalline PLLA is 93.7 J·g^−1^.

The water contact angles of all samples were measured with a contact angle analyzer (SDC-350; Dongguan Shengding Precision Instrument Co., Ltd., Dongguan, China) at room temperature.

### 2.4. Biocompatibility Test

The specimen was prepared as follows. The sample (HAP, ESOA-HAP, or OPLA-ESOA-HAP), prepared in Section 2.2, was initially mixed with ethanol to form a suspension with a concentration of 1.0 mg/mL^−1^. Subsequently, the suspension was carefully applied onto a clean cover slide (24 × 24 mm) and left to air-dry at 25 °C. For subsequent biocompatibility testing, the specimen collected using this method undergoes sterilization through exposure to UV radiation for a duration of 30 min.

#### 2.4.1. Cultivation of the MSCs

Wistar rat mesenchymal stem cells (MSCs) were provided by the National Collection of Authenticated Cell Cultures. The identifier of this cell was CSTR:19375.09.3101RATSCSP401. These MSC cells were cultured in a flask containing DMEM/F12 = 1:1 (Hyclone) and maintained at 37 °C in a humidified incubator with a 5% CO_2_ atmosphere. The cell culture density was maintained at 2.0 × 10^4^ cells·cm^−3^. The culture medium was replaced every three days. Once cell coverage reached approximately 80–90% on the flask surface, a trypsin solution (2.5 mg·mL^−1^) and an EDTA solution (0.2 mg·mL^−1^), mixed in equal volumes, were used to detach adherent cells from the task. The cells were subjected to three washes with a 0.1 mol·mL^−1^ phosphate-buffered solution (PBS) and subsequently centrifuged at 1000 rpm for five minutes. Wistar rat MSC cells were suspended in the medium at a cell density of 1.0 × 10^5^ cells per milliliter.

The cell viability was evaluated using the 3-(4,5-dimethylthiazol-2-yl)-2,5-diphenyltetrazolium bromide (MTT) assay. Then, 200 μL of HAP, ESOA-HAP, or OPLA-ESOA-HAP at a concentration of 20 mg·mL^−1^ was added to individual wells of a 96-well plate and dried under an infrared lamp in a laminar flow clean bench. Wistar rat MSC cells were then seeded into these wells at a density of 1.0 × 10^4^ cells·cm^−2^. After synchronization, the cells were incubated with HAP, ESOA-HAP, and OPLA-ESOA-HAP for 24, 48, and 72 h. Subsequently, the medium in each well was aspirated and replaced with a solution of MTT (5 mg·mL^−1^). Prior to this step, the cells were rinsed with 200 mL of PBS per well.

After a two-hour incubation at 37 °C, the MTT solution in each well was aspirated and replaced with 150 mL of dimethylsulphoxide (DMSO) to dissolve formazan crystals. The plate was agitated, and the optical density (OD) at 570 nm for each well was measured using a Tecan multiplate reader (XDS-18, Chongqing, China). The absorbance associated with control cells was used as the viability value of 100%. The inhibition rate was determined using Equation (2).

Inhibition rate = [1 − (A_2_ − A_0_)/(A_1_ − A_0_)] × 100% (2)

A_0_: The absorbance of the well with no material in it;
A_1_: The absorbance of the control well;
A_2_: The absorbance of the experimental group.

#### 2.4.2. The Adhesion Test of MSCs on the Materials

The MSC cells were seeded onto cover slides coated with HAP, ESOA-HAP, and OPLA-ESOA-HAP, respectively, at a density of 1.0 × 10^5^ cells·well^−1^ (Costar, Corning, NY, USA) in 6-well cell culture plates. Subsequently, 3 mL of medium was added to each well and combined with the MSCs for a cultivation period of 45 min. Following 3 times of rinsing with PBS, the cover slides were fixed for 8 min using a solution of 3% glutaraldehyde in PBS. After thorough washing, crystal violet (0.1 mL, 9%) was employed for cellular staining. The OD at 570 nm for each well was determined using a Tecan multiplate reader (XDS-18, Chongqing, China).

The cell adhesion evaluation was conducted following the aforementioned protocol, with the exception that cells were cultivated with the sample for 24 h. Subsequently, the cell attachment and morphology of various samples were examined using a reverse microscope (TE2000U, NIKON, Tokyo, Japan). Nine photographs capturing different positions on the cover slide were then captured using a digital camera (DXM1200F).

### 2.5. Mechanical Test

The properties of tensile strength, tensile modulus, breaking elongation, bending strength, and bending modulus were measured in accordance with the GB/T 1040.2-2022 standard (China) [20] using an electromechanical universal testing machine (CMT6000, Sans, Shenzhen, China). Each specimen was evaluated using 5 parallel samples, and the mean values along with their corresponding standard error were provided.

## 3. Results and Discussion

### 3.1. Materials Characterization

**Figure 1:**
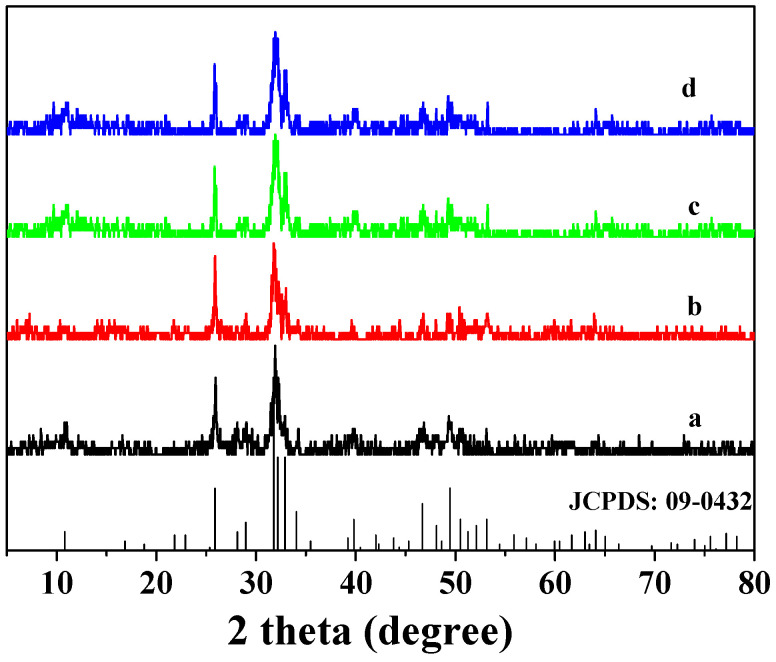
XRD spectra of HAP (**a**), ESOA-HAP (**b**), OPLA-HAP (**c**), and OPLA-ESOA-HAP (**d**).

The XRD patterns of HAP, ESOA-HAP, OPLA-HAP, and OPLA-ESOA-HAP are presented in Figure 1. These synthetic samples exhibit similar characteristic diffraction patterns and exhibit diffraction patterns that are consistent with hexagonal Ca_10_(PO_4_)_6_(OH)_2_ in the P63m space group (JCPDS: 09-0432). Upon the modification of HAP, the major diffraction peaks, including (100), (002), (211), (112), (300), (202), (310), (222), (213), and (004), remain virtually unchanged. This observation suggests that the modification does not alter the crystal structure of HAP.

**Figure 2:**
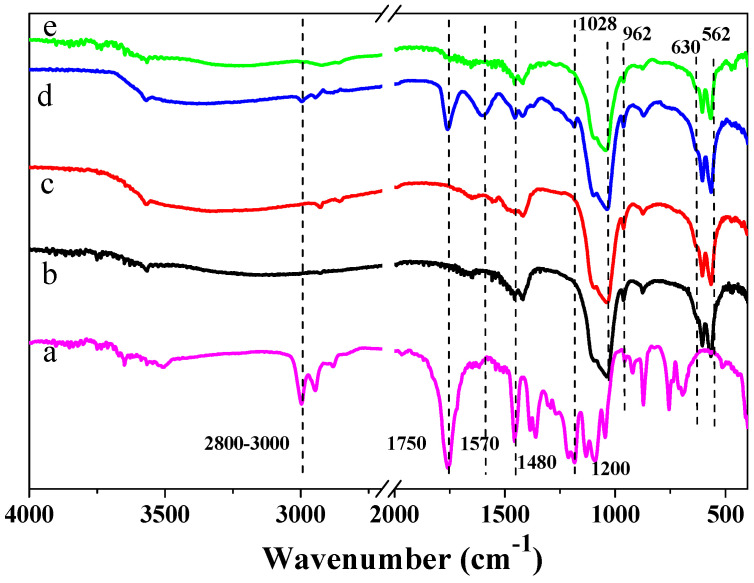
FT-IR spectrum of the OPLA (**a**), HAP (**b**), ESOA-HAP (**c**), OPLA-ESOA-HAP (**d**), and OPLA-HAP (**e**).

The FT-IR results of OPLA, HAP, OPLA-HAP, and OPLA-ESOA-HAP are presented in Figure 2. The peaks observed at 962 and 1028 cm^−^^1^ correspond to the stretching vibrations attributed to the phosphate group (PO43−), while the peaks detected at 562 cm^−^^1^ and 603 cm^−^^1^ could be ascribed to the deformation vibrations of PO43−. The constancy of these peaks across all samples provides compelling evidence that the structural integrity of HAP remains unaltered following its adjustment.

The peak observed at 2800–3000 cm^−^^1^ in the spectra of ESOA-HAP and OPLA-ESOA-HAP is attributed to the vibration of the methylene group and the methyl group on the surface of OPLA-ESOA-HAP. Additionally, the ester group vibration in the OPLA is assigned to the prominent adsorption peak at 1750 cm^−^^1^. The adsorption peaks observed at 1570 cm^−^^1^ and 1480 cm^−^^1^ in the OPLA-ESOA-HAP spectra could be attributed to the vibration of the carboxyl group in the calcium carboxylate. Furthermore, the weak adsorption at 1200 cm^−^^1^ is ascribed to the C-O vibration in OPLA.

The FT-IR spectra revealed the formation of an ionic interaction between the carboxyl group of ESOA and the Ca^2+^ on the surface of HAP. In contrast, none of the four peaks (1200 cm^−^^1^, 1480 cm^−^^1^, 1570 cm^−^^1^, and 1750 cm^−^^1^) were observed in the OPLA-HAP spectrum, indicating that the direct grafting of OPLA onto HAP was not feasible under the given reaction conditions. The use of ESOA was crucial for modulating HAP.

**Figure 3:**
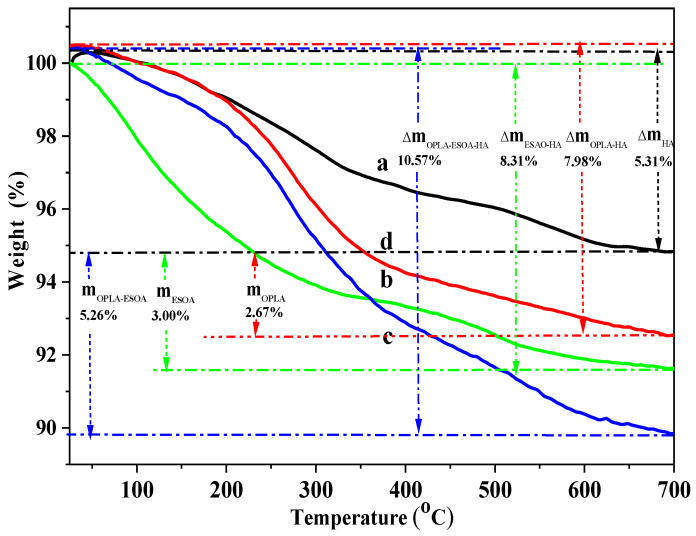
TGA curves of n-HA (**a**), ESBO-HA (**b**), OPLA-ESBO-HA (**c**), and OPLA-HA (**d**).

The thermogravimetric analysis (TGA) curves of HAP, ESOA-HAP, OPLA-HAP, and OPLA-ESOA-HAP are shown in Figure 3. The graft amounts of these materials are presented in Table 1. Upon increasing the temperature from 25 °C to 700 °C, distinct weight losses were observed for all the samples. The mass of the HAP sample for the unmodified HAP sample gradually dropped until a weight loss of 5.31% was achieved. The weight loss for the ESOA-HAP sample is 8.31, of which 3.1% is due to the breakdown of epoxidized soybean oil. For the OPLA-ESOA-HAP sample, the weight loss is 10.57 wt%, among which 2.26% was from the decomposition of OPLA. Through co-precipitation, the ESOA grafted onto the HAP surface. The results of XRD, FTIR, and TGA showed that the OPLA connected to ESOA-HAP successfully.

**Figure 4:**
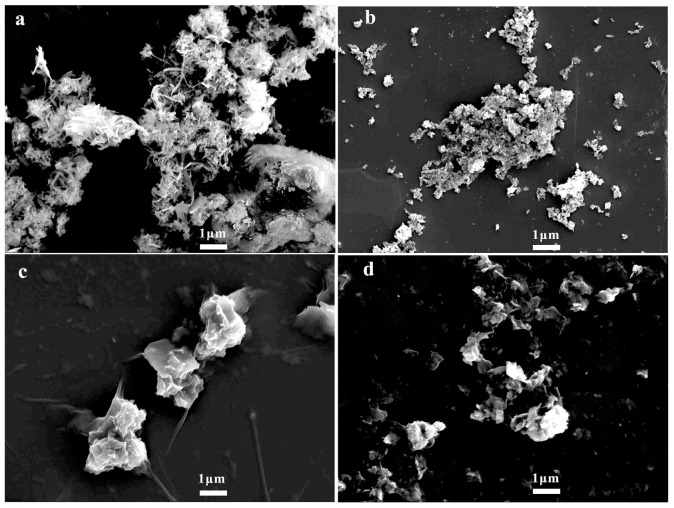
SEM images of HAP (**a**), ESOA-HAP (**b**), OPLA-HAP (**c**), and OPLA-ESOA-HAP (**d**).

The scanning electron microscope (SEM) images of HAP, OPLA-HAP, ESOA-HAP, and OPLA-ESOA-HAP are presented in Figure 4. In Figure 4a, the HAP particles exhibit an aggregated morphology. It can be observed that the grafting amount of ESOA is relatively low in these samples; however, the grafting of ESOA does decrease its aggregation to some extent. Conversely, both OPLA-HAP and OPLA-ESOA-HAP demonstrate significantly improved dispersity, as shown in Figure 4c,d. When grafted with ESOA and OPLA, HAP particles show minimal aggregation along with an overlaying layer composed of OPLA polymers. To evaluate the presence of polymers on the surface of OPLA-ESAO-HAP, samples were evenly dispersed onto a silicon plate followed by elemental analysis through an EDS line scan. The results confirm the existence of carbon atoms on particle surfaces, which is further supported by the statistical data presented in Figure S1 and Table S1, indicating relatively substantial levels of organic material (OPLA). Collectively, these findings provide strong evidence supporting the successful achievement of grafting involving both ESOA and OPLA onto HAP’s surface.

**Figure 5:**
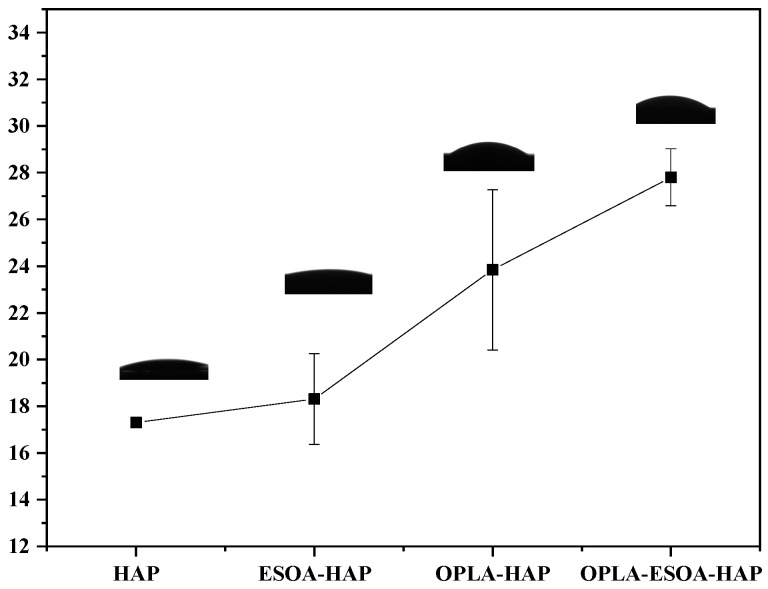
Water contact angle of HAP, ESOA-HAP, OPLA-HAP, and OPLA-ESOA-HAP.

A comprehensive investigation was conducted to examine the wettability of four distinct particle types, and the corresponding results are illustrated in Figure 5. These findings unequivocally demonstrate that surface modification effectively enhances the hydrophobicity of HAP, as evidenced by the significantly augmented contact angle (27.8 ± 1.22°) observed for OPLA-ESOA-HAP, surpassing that of all other samples.

### 3.2. The Biocompatibility of the HAP and Modified HAP

Biocompatibility is a crucial property for bioresorbable materials, particularly in the context of bone tissue engineering, where the transformation of mesenchymal stem cells (MSCs) into osteoblasts holds significant importance [21,22]. The success of an MSC/biomaterial composite for osteogenesis may rely on the survival ability and initial adherence capability of MSCs to the biomaterial. Ensuring a high viability and strong adhesion of MSCs onto the biomaterial is crucial for subsequent differentiation and growth of new bones.

**Figure 6:**
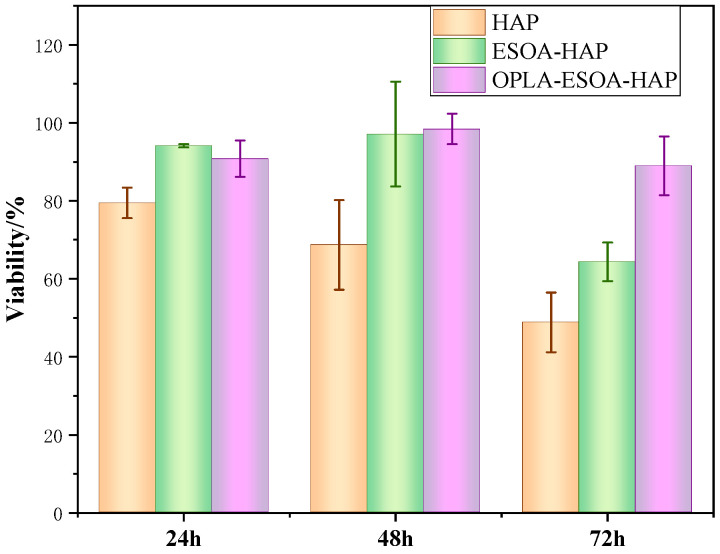
Viability of the MSC cells co-cultured with HAP, ESOA-HAP, and OPLA-ESOA-HAP at different time intervals. The concentration of the samples are 1000 μg·mL^−^^1^.

The biocompatibility of the synthesized materials was characterized by conducting a cell adhesion experiment using MSCs from Wistar rats in this study. A commercially available MTT assay was employed to determine the cell viability of the HAP, ESOA-HAP, and OPLA-ESOA-HAP at different time intervals, as shown in Figure 6. The viability of MSCs co-cultured with HAP exhibited a decrease of 30.6% as the incubation time extended from 24 h to 72 h. The viability of MSCs co-cultured with OPLA-ESOA-HAP remained above 89% throughout various culture intervals, which is comparatively higher than that observed for ESOA-HAP and HAP. It should be noted that the concentration of OPLA-ESOA-HAP applied in this work (1.0 mg·mL^−^^1^) exceeds the typical concentration reported in the literature (usual 0.1 mg/mL) [23,24]. These findings suggest that incorporating ESOA and OPLA onto the HAP surface significantly increases its biocompatibility.

**Figure 7:**
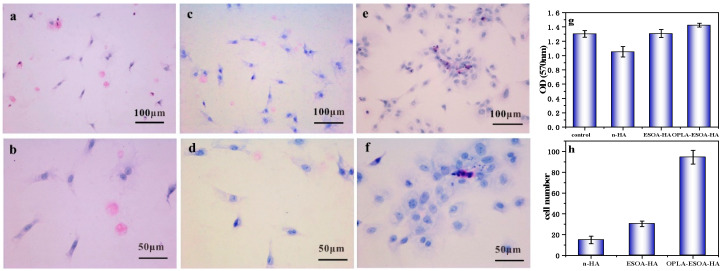
The cells adhesion experiments of the samples. Optical micrographs of HAP (**a**,**b**), ESOA-HAP (**c**,**d**), and OPLA-ESOA-HAP (**e**,**f**); the MSCs are cultivated with the samples for 24 h (**a**–**f**). OD of the different sample after cultivated for 45 min (**g**). The statistical number of the cells in the image for 100 μm (**h**). The red particles are these HA and modified HA particles. The blue one presents the cells stained by crystal violet.

The optical microscope images in Figure 7a–f depict the cellular adherence to the materials (HAP, ESOA-HAP, and OPLA-ESOA-HAP). The initial optical density (OD) after cultivating for a 45 min cultivation period is presented in Figure 7g. Statistical data regarding cell numbers are displayed in Figure 7h. As illustrated in Figure 7a–d, HAP particles exhibit minimal cell adhesion, whereas an increased number of cells can be observed surrounding ESOA-HAP particles. A substantial number of MSCs adhered to the particles in the OPLA-ESOA-HAP group, with a cell count of 94, nearly three times higher than that of ESOA-HAP and six times greater than that of HAP. Figure 7g demonstrates that the OD of the OPLA-ESOA-HAP sample was higher compared to HAP and ESOA-HAP after a cultivation period of 45 min. The results demonstrate a higher adhesion of MSCs on the OPLA-ESOA-HAP composite with just 45 min of contact. Incorporating ESOA into HAP slightly enhances cell adhesion to MSCs, while the addition of OPLA significantly improves both cell survival and adhesion on these materials.

Based on the results of the biocompatibility test, the addition of ESOA and OPLA significantly enhances the vitality and adhesion of OPLA-ESOA-HAP to MSCs, potentially attributed to their increased cellular contact sites. The sample from OPLA-ESOA-HAP exhibited superior adhesion to MSCs compared to other samples, as depicted in Figure 7e,f. These findings demonstrate that polydopamine (p-DA)-coated HAP effectively enhances cell attachment on polymer-based cement by providing a side-range surface for peptide conjugation [25]. In this work, the ESOA and OPLA could serve a similar function to the p-DA by providing a more active surface for cell attachment. The OPLA-ESOA-HAP composite exhibits promising bioceramic properties for bone fixation materials due to its favorable cell adhesion properties and low toxicity. Given that MSCs demonstrate high viability on this material and exhibit strong adhesion to its surface, these properties collectively contribute to promoting new bone formation in the initial stage.

### 3.3. Mechanical Properties

In previous studies, the modified HAP was blended with PLLA through co-precipitation in a solution or an internal mixer at 170 °C. Subsequently, the resulting composites were subjected to hot pressing above 10 MPa to achieve a regular shape [26,27,28]. The composites prepared through these blending and shaping methods exhibited relatively high mechanical properties. However, irregular shapes, such as the screw or plate used for bone fixation, render them somewhat unsuitable for the industrial manufacture of internal fixation device. On the contrary, the twin crew extrusion and injection molding method is considered to be a suitable blending and shaping technology for the industrial manufacture of the HAP/PLLA composite. However, it should be noted that the mechanical strength may decrease during the manufacturing process due to the thermal degradation of the polymer caused by prolonged exposure under high temperatures. In this study, we fabricated HAP/PLLA samples by incorporating OPLA-ESOA-HA into the PLLA matrix through twin-screw extrusion and injection molding techniques to assess the mechanical properties of these blended composites.

**Figure 8:**
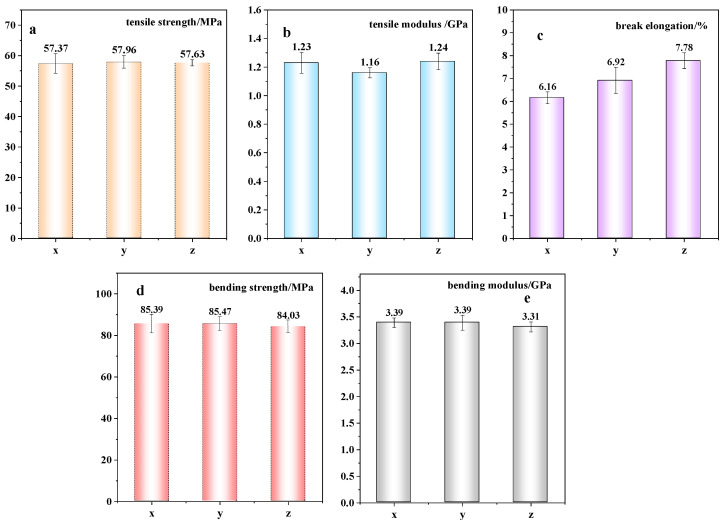
Effect of the filler kind on the mechanical properties: (**a**) tensile strength, (**b**) tensile modulus, (**c**) break elongation, (**d**) bending strength, and (**e**) bending modulus. (**x**) HA/PLLA, (**y**) OPLA-ESOA-HA/PLLA, (**z**) OPLA-ESOA-HA/PLLA, The filler content is 5 wt%.

The results of the mechanical properties of the three types of blended composites are presented in Figure 8. It can be observed that there were no significant differences in the tensile strength (Figure 8a), tensile modulus (Figure 8b), bending strength (Figure 8d), and bending modulus (Figure 8e) among the HAP/PLLA, ESOA-HAP/PLLA, and OPLA-ESOA-HAP/PLLA composites. The tensile strength was all above 57 MPa, while the bending strength values for all three composites exceeded 84 MPa, which is relatively high compared to some previous studies [29,30]. The break elongation (Figure 7c) of HAP/PLLA, ESOA-HAP/PLLA, and OPLA-ESOA-HAP/PLLA is 6.16%, 6.92%, and 7.78%, respectively. The observed increase in break elongation for all three blended composites aligns with our design objectives. The introduction of organic polymer and ESOA modified on the HAP surface promotes entanglement with the PLLA matrix, leading to a debonding effect during the mechanical testing and, consequently, an increase in break elongation. Additionally, the ESOA incorporated in the blended composites could act as plasticizer in the preparation process and enhanced its break elongation. These findings suggest that the introduction of OPLA and ESOA did not adversely affect the mechanical properties but rather slightly improved the ductility of the composites.

**Figure 9:**
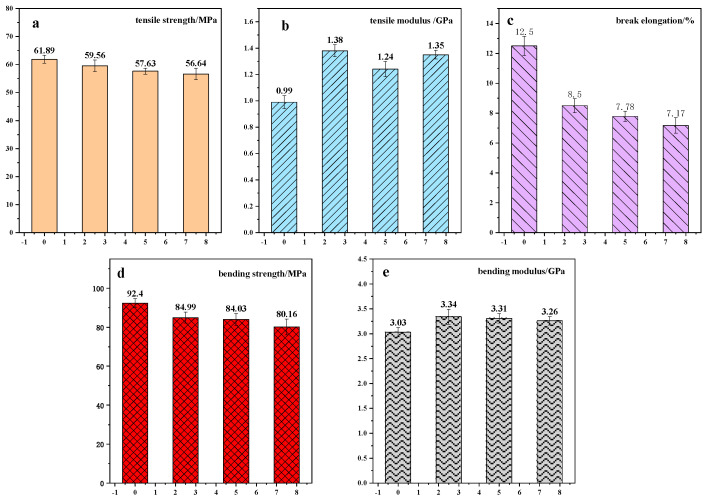
The effect of OPLA-ESOA-HAP amount on the mechanical properties of composite. (**a**) tensile strength, (**b**) tensile modulus, (**c**) break elongation, (**d**) bending strength, and (**e**) bending modulus.

The influence of the filler content on the mechanical properties is illustrated in Figure 9. In this figure, the tensile strength (Figure 9a), tensile modulus (Figure 9b), break elongation (Figure 9c), bending strength (Figure 9d), and bending modulus (Figure 9e) of the OPLA-ESOA-HAP/PLLA composite with varying amounts of OPLA-ESOA-HAP filler (0.0 wt%, 2.5 wt%, 5.0 wt%, and 7.5 wt%) are presented. The tensile strength of the pure PLLA (0 wt% filler content) was measured to be 61.89 MPa, slightly surpassing that of the composites with 2.5 wt%, 5.0 wt%, and 7.5 wt% filler contents. Among the three composites filled with OPLA-ESOA-HAP, the highest tensile strength of 59.56 MPa was observed in the composite containing a 2.5 wt% filler. The tensile strength decreases as the filler content increases, possibly due to the aggregation of the filler particles. In composites with large filler particles, stress concentration may occur under a mechanical load. The tensile strength is slightly lower than that of pure PLLA due to the absence of the screw extrusion process under relatively high temperatures with a filler. When the filler amount is 7.5 wt% in this work, the tensile strength of OPLA-ESOA-HAP/PLLA exceeds 56.0 MPa, surpassing the values reported in previous studies [29,30,31]. According to Figure 9c,d, the break elongation and bending strength exhibit a similar trend to tensile strength. This phenomenon can be attributed to the aggregation of modified filler. Fortunately, an increase in the amount of filler leads to an enhanced tensile modulus and bending modulus. Both the tensile and bending moduli increased proportionally with increasing filler amounts. The OPLA-ESOA-HAP/PLLA composite exhibited the highest tensile modulus and bending modulus at a filler content of 2.5 wt%, surpassing that of PLLA. The mechanical properties of these composites did not exhibit a significant increase with increasing filler content; however, relatively favorable mechanical properties can be achieved after subjecting the materials to two high-temperature manufacturing processes.

**Figure 10:**
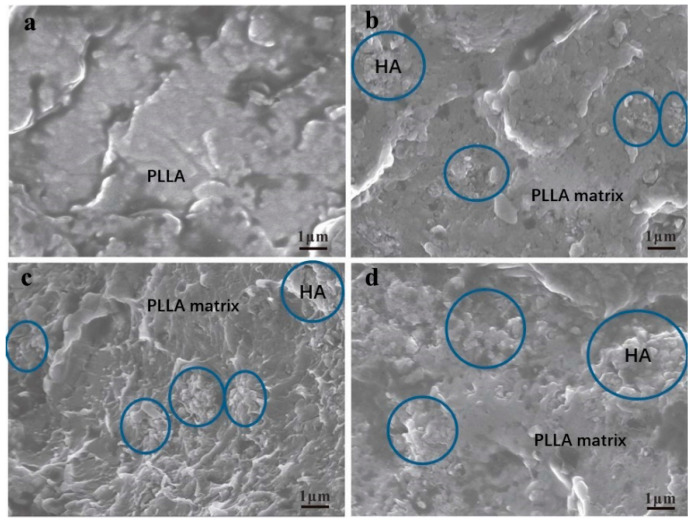
The SEM image of the fracture of the composites PLLA (**a**); HAP/PLLA (**b**); ESOA-HAP/PLLA (**c**); and OPLA-ESOA-HAP (**d**). The content of the filler in the composite (**b**–**d**) was 5 wt%.

The SEM image in Figure 10 illustrates the fracture of the PLLA, ESOA-HAP/PLLA, and OPLA-ESOA-HAP/PLLA composites. It is evident that the fracture region of the composite exhibits a relatively smooth surface. No notable presence of large, aggregated HAP particles, holes, or cracks was observed. The incorporation of OPLA-ESOA-HAP particles into the PLLA matrix did not result in the formation of larger aggregates, indicating that OPLA-ESOA-HAP serves as an effective filler for preparing PLLA-based composites using screw extrusion and injection molding processes.

The DSC curves of the three composites are presented in Figure S2. Table 1 displays the thermal characteristics of HAP/PLLA, ESOA-HAP/PLLA, and OPLA-ESOA-HAP/PLLA, as determined by DSC analysis. It is observed that the addition of OPLA-ESOA-HAP particles to the PLLA composite leads to a slight increase in the glass transition temperature (T_g_). The order of crystallization temperature (T_c_) and crystallinity (X) for the three composite is as follows: HAP/PLLA < ESOA-HAP/PLLA < OPLA-ESOA-HAP/PLLA. According our previous research, incorporating inorganic rigid fillers into the composite leads to an increase in its T_g_ [32]. Although it is expected that the incorporation of ESOA would lead to a decrease in the T_g_ of the composite [16], our experimental results demonstrate that the T_g_ of OPLA-ESOA-HAP/PLLA remains unchanged. Firstly, the content of ESOA in the OPLA-ESOA-HAP is about 3 wt%, which is relatively low. Additionally, due to its connection with OPLA and HAP, ESOA exhibits restricted mobility within the matrix and cannot functional plasticizer.

**Scheme 1:**
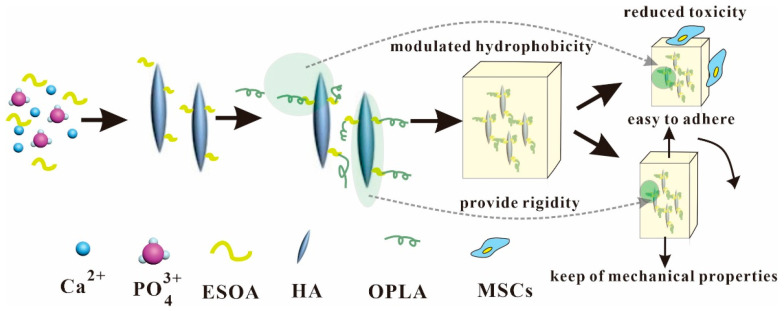
The probable modification mechanism of the composite.

The proposed modification mechanism is illustrated in Scheme 1. The significantly enhanced biocompatibility of OPLA-ESOA-HAP, along with its relatively high mechanical properties, renders it a promising bioresorbable material for bone fixation and repair.

## 4. Conclusions

The present study investigates the surface modification of HAP using ESOA, followed by grafting OPLA. By means of a co-precipitation reaction involving CaCl_2_, Na_3_PO_4_, and ESOA, additional active groups are introduced onto the surface of HAP. Subsequently, the surface of ESOA-HAP is grafted with OPLA. The hydrophilicity of this modified OPLA-ESOA-HAP was reduced, leading to an enhanced wettability of these particles. It is noteworthy that the introduction of OPLA-ESOA-HA significantly enhances both the viability and cell adhesion of MSCs compared to unmodified HAP. The utilization of HAP/PLLA, ESOA-HAP/PLLA, and OPLA-ESOA-HAP/PLLA composites prepared via melt extrusion and injecting mold is advantageous for osteogenesis in the initial stage. However, the incorporation of OPLA and ESOA onto the surface of HAP has minimal impact on the mechanical properties of the composite. The incorporation of OPLA-ESOA-HAP resulted in a slight enhancement in the ductility of the OPLA-ESOA-HAP/PLLA composite. The excellent biocompatibility exhibited by OPLA-ESOA-HAP, coupled with the relatively high mechanical properties when combined with PLLA, renders it a promising filler for the bioresorbable material.

## Figures and Tables

**Table 1:** Thermal properties of three kinds of HA/PLLA composites.

Entry	T_g_/°C	T_c_/°C	T_m_/°C	ΔH_m_/J/g	X/%
HAP/PLLA	54.37	104.87	165.69	36.58	39.4
ESOA-HAP/PLLA	54.50	108.36	166.20	35.16	37.5
OPLA-ESOA-HAP/PLLA	55.76	109.14	166.73	32.94	35.2

T_g_: glass transition temperature; T_c_: crystallization temperature; T_m_: melting temperature; ΔH_m_: melting enthalpy; X: crystallinity. The filler content is 5 wt%.

## Data Availability

There is no new data were create.

## Supplementary Materials

The following supporting information can be downloaded at: https://www.mdpi.com/article/10.3390/ma17112620/s1. Figure S1: The line scan image of the OPLA-ESOA-HA and the element dispersity of different positions. Table S1: The element amount of the particle measured by EDS. Figure S2: DSC curves of n-HAP/PLLA, ESOA-HAP/PLLA, and OPL-ESOA-HAP/PLLA.
