# Supplementary material for: Epoxidized Soybean Oleic Acid/Oligomeric Poly(lactic acid)-Grafted Nano-Hydroxyapatite and Its Role as a Filler in Poly(L-lactide) for Potential Bone Fixation Application
Source: Materials (Basel). 2024 May 29;17(11):2620. doi: 10.3390/ma17112620 (PMC11173816; doi:10.3390/ma17112620)
Supplement: Supplementary file 1 [file materials-17-02620-s001.zip › materials-2983770-supplementary.docx]

Article

Epoxidized Soybean Oleic Acid/Oligomeric Poly (Lactic Acid)-Grafted Nano-Hydroxyapatite and Its Role as a Filler in
Poly (L-Lactide) for Potential Bone Fixation Application

Chen Huang ^1,†^, Xin-Yu Luo ^1,†^, Zi-Sheng Chao ^1^, Yue-Fei Zhang ^1^, Kun Liu ^2^, Wen-Jun Yi ^1,^*, Li-Jun Li ^2,^*
and Zeyan Zhou ^3,^*

^1^ College of Materials Science and Engineering, Changsha University of Science &Technology,
Changsha 410082, China; 19375178158@163.com (C.H.); 19308467138@163.com (X.-Y.L.);
chao_zs@aliyun.com (Z.-S.C.); zhangyuefei@csust.edu.cn (Y.-F.Z.)

^2^ College of Chemistry and Chemical Engineering, Hunan Institute of Science and Technology,
Yueyang 414006, China; liukun328@126.com

^3^ College of Materials Science and Engineering, Hunan University, Changsha 410012, China

***** Correspondence: yiwenjun8933@163.com (W.-J.Y.); lilijun115@163.com (L.-J.L.);
zhouzeyan@hnu.edu.cn (Z.Z.)

^†^ These authors contributed equally to this work.

Supplementary results

|  |  |
| --- | --- |
| **(a)** | **(b)** |

**Figure S1.** The line scan image of the OPLA-ESOA-HA and the element dispersity of different positions.

**Table S1.** The element amount of the particle measured by EDS.

| **Element** | **Wt %** | **Weight %** | **Atomic %** | **Error %** |
| --- | --- | --- | --- | --- |
| C K | 17.79 | 20.78 | 34.74 | 15.21 |
| O K | 24.51 | 28.63 | 35.92 | 15.15 |
| P K | 23.25 | 27.16 | 17.61 | 2.35 |
| Ca K | 20.05 | 23.42 | 11.73 | 2.95 |

**Figure S2.** DSC curves of n-HAP/PLLA, ESOA-HAP/PLLA, and OPL-ESOA-HAP/PLLA.
